# Supplementary material for: Evaluating the predictive accuracy of cognitive screeners BAMCOG and MoCA in identifying postoperative delirium risk in aortic valve replacement patients: A cohort study
Source: PLOS Digit Health. 2025 Sep 8;4(9):e0001005. doi: 10.1371/journal.pdig.0001005 (PMC12416729; doi:10.1371/journal.pdig.0001005)
Supplement: Attachment — Submitted filename: Rebuttal letter.docx [file pdig.0001005.s002.docx]

Dear Peter H Charlton,

Thank you very much for your message and for the constructive feedback provided by you and the reviewer on our manuscript entitled *Evaluating the predictive accuracy of cognitive screeners BAMCOG and MoCA in identifying postoperative delirium risk in aortic valve replacement patients: A cohort study.* We greatly appreciate the time and effort that you and the reviewer have invested in evaluating our work.

We have carefully considered all comments and have revised the manuscript accordingly. In the attached table, we respond to each comment individually, outlining the changes made or, where applicable, providing a rationale for not implementing a specific suggestion.

We hope that the revised manuscript meets your expectations and we look forward to your further evaluation.

Sincerely,
Mariska te Pas
On behalf of all authors

**Editor**

| **Comment** | **Revised yes/no** | **Action** |
| --- | --- | --- |
| 1. Should the abbreviation for postoperative encephalopathy be POE instead of the PAE currently used in the manuscript? | Yes | We agree with the editor that using **POE** (postoperative encephalopathy) is more appropriate and consistent, particularly since we use **POD** for postoperative delirium elsewhere in the manuscript. We have therefore replaced all instances of "PAE" with "POE" throughout the text. |
| 2. The first line of the results is, in my view, confusing. Currently it reads: "The sample comprised 9 women...". I initially thought this meant that there were a total of 9 study participants, whereas actually there were 50 participants, 9 of whom were women. Please consider re-phrasing this. | Yes | We have rephrased the sentence to improve clarity. It now reads: **“The study sample consisted of 50 participants, including 9 women.”** |
| 3. Figure 1: Should the title read "ROC Prediction of PAE" instead of "ROC Prediction of AE"? | Yes | We have adapted the title to **"ROC Prediction of POE"** |
| 4. Figures 1 and 2: Whilst I appreciate the shaded areas provide information on confidence intervals (I presume), in this particular case I would suggest you consider leaving out the shaded areas. In my view they add little information as it is difficult to discern the individual areas, they make it more difficult to see the ROC curves, and they may even be misleading because there are more shading colours than ROC curves (due to merging colours in the overlapping areas) which places emphasis on the overlapping areas rather than the individual areas. | Yes | We fully agree with the editors assessment. We had experimented with various options to display the confidence intervals clearly, but as noted, the overlapping shaded areas remained visually confusing and detracted from the clarity of the ROC curves. We have therefore removed the shaded areas from Figures 1 and 2 in the revised manuscript to improve interpretability. |
| 5. Section 2.2.1 states that different versions of MoCA were used at various measurement points. Please could you clarify whether the different measurement points refer to: (i) different days (e.g. day 1, 14, 15, 16, ... as illustrated in Table 1); or (ii) changes during the overall study (e.g. the first 25 participants used one version, and the next 25 participants used a different version). Please also briefly state / discuss whether or not the use of different versions could impact the results and conclusion. | Yes | We thank the editor for pointing this out and agree that clarification was needed. We have revised the manuscript to explicitly state which MoCA versions were used at which time points. Specifically, the Dutch MoCA version 8.1 was administered preoperatively and again on postoperative day 7. Version 7.2 was used on postoperative day 1, and version 7.3 on postoperative day 3. Besides, we have added a brief statement to the manuscript discussing the potential impact of using different MoCA versions. Specifically, we note that different versions were intentionally used to minimize learning effects associated with repeated testing. Although minor variations exist between versions, all are considered equivalent in difficulty and scoring, and therefore unlikely to have affected the overall results or conclusions of the study. This information has been added to Section 2.2.1 of the revised manuscript. |
| 6. In the discussion please discuss any previous work reporting the accuracy of MoCA and BAMCOG in predicting POD and PAE. | Yes | To our knowledge, no previous studies have specifically evaluated the predictive accuracy of the BAMCOG or the MoCA in relation to postoperative delirium (POD) or postoperative acute encephalopathy (POE). However, prior research has investigated the association between general preoperative cognitive functioning and the risk of developing POD. This broader literature is discussed in Section 4.1 of the revised manuscript. We have now added a statement to clarify that our study represents, to our knowledge, the first evaluation of the predictive value of both the BAMCOG and the MoCA for POD and PAE. |
| 7. Section 2.3 states that a "second question examines ICU and hospital stay durations". However, I didn't see this question addressed in the text in the results section. Whilst results on this are provided in Table 3, I think if this is one of two questions addressed by the study then it should be referred to in the results text, rather than waiting until the Discussion section. More generally, I have concerns about this being a key question of the study: (i) I don't know whether the apparent difference in length of stay is statistically significant as no statistical tests are provided in Table 3; and (ii) I don't know whether this is due to differences in POD status, or other unrelated differences between the groups (again, I think statistical tests would help address this concern). A simpler way to address this concern might be to: (i) not state that this is a main question of the research in Section 2.3; and (ii) to simply state this as an observation that length of stay was lower in the non-POD group, and remove the emphasis on it by not stating it in the first paragraph of the results. | Yes | We agree that the emphasis on ICU and hospital length of stay as a central research question was not sufficiently supported by the data and statistical analysis in its previous form. In response, we have removed the reference to this question as a main aim of the study from Section 2.3. Additionally, the observed differences in length of stay between the POD and non-POD groups are now briefly noted as a descriptive observation at the end of the Results section, in alignment with Table 3, without overstating their interpretive value. We appreciate the reviewer’s suggestion, which helped clarify the focus of the manuscript. |

**Reviewer 1**

| **Comment** | **Revised yes/no** | **Action** |
| --- | --- | --- |
| 1. Expand discussion on limitations of DeltaScan and DOS as non-gold standard tools. While practical, their diagnostic sensitivity and specificity can vary. | Yes | We agree with the reviewer and have expanded the limitations section accordingly. While DeltaScan and DOS are practical tools, neither qualifies as a diagnostic gold standard. We now also note that the DOS may be less sensitive to hypoactive delirium, as suggested by previous studies. |
| 2.Clarify whether BAMCOG results were adjusted for confounders like educational level or digital literacy—factors that can impact game-based performance. | Yes | We have added a paragraph to the Discussion section addressing the potential influence of digital literacy on BAMCOG performance. In this addition, we also refer to studies by Wu et al. and Bayer et al., which demonstrate that tablet-based cognitive tests and digital cognitive assessment tools are valid, feasible, and usable among older adults. |
| 3. The sample size, while justified, limits generalizability. The authors mention this, but future power calculations could be more detailed. | Yes | We have now added a brief sample size calculation to the Recommendations section. This outlines how a sample size of approximately 230 patients would allow for reasonable estimation of sensitivity and specificity, based on expected values and delirium prevalence. |
| 4. Add brief detail on usability: The manuscript states the usability study is submitted elsewhere. Consider including a summary statistic or SUS score in this paper. | No | Thank you for this suggestion. Usability was assessed using the System Usability Scale (SUS), and the resulting score of 79.7 is reported in Section 4.2 of the Discussion. |
| 5. A table summarizing key results (AUROC, CI, p-values) would help readers quickly interpret performance of BAMCOG vs MoCA. | Yes | To improve clarity and accessibility of the key findings, we have added **Table 4**, which summarizes the predictive performance and logistic regression results for MoCA and BAMCOG, including AUROC values, confidence intervals, and p-values. |
| **Minor Edits** |  |  |
| 1. Typo: “Postoperative Delirium(N=36)” → add a space. | Yes | We added a space. |
| 2. Abbreviations like “IE” for alcohol units should be clearly defined on first use. | No | The abbreviation “IE” is first used in Table 2 and is defined in the table legend as referring to "units of alcohol" |
